# Frequency of Polyomavirus BK Infection in Kidney Transplant Patients Suspected to Nephropathy

**Published:** 2015-05-01

**Authors:** M. Pakfetrat, R. Yaghobi, Z. Salmanpoor, J. Roozbeh, S. Torabinezhad, S. Kadkhodaei

**Affiliations:** 1 Department of Internal Medicine, Shiraz Nephro-Urology Research Center, Shiraz University of Medical Sciences, Shiraz, Iran; 2 Shiraz Transplant Research Center, Shiraz University of Medical Sciences, Shiraz, Iran; 3 Department of Internal Medicine, Shiraz University of Medical Sciences, Shiraz, Iran; 4 Pathology Department, Shiraz Nephrology-Urology Research Center, Shiraz Medical School, University of Medical Sciences, Shiraz, Iran

**Keywords:** Polyomavirus BK, Nephropathy, Kidney transplantation

## Abstract

**Background:**

Polyomavirus BK is a major cause of nephropathy in immunosuppressed transplanted patients. Non-invasive diagnostic protocols such as molecular detection of polyomavirus BK replication are a useful strategy to predict BK virus-associated nephropathy (BKVAN).

**Objective:**

To determine the prevalence of polyomavirus BK infection among kidney transplant patients suspected to have BKVAN.

**Methods:**

In a cross-sectional study 108 kidney transplanted patients whose laboratory and clinical presentation were in favor of nephropathy between 2010 and 2012, were enrolled for analysis. Polyomavirus BK replication was evaluated in plasma and tissue samples of studied patients using a quantitative real-time PCR. Active cytomegalovirus infection was analyzed in studied patients using antigenemia method. A possible association between polyomavirus BK infection with clinical and laboratory risk factors of BKVAN were evaluated.

**Results:**

The polyomavirus BK replication was found in 17 (15.7%) of 108 of plasma and 9 (11%) of 82 tissue samples in kidney transplanted patients. Cytomegalovirus co-infection was found in 3 of 17 and 3 of 9 plasma and tissue samples in polyomavirus BK infected patients, respectively. Significant associations were found between polyomavirus BK infection with tubulointerstitial nephritis and acute cellular rejection, as important pathologic findings of BKVAN.

**Conclusion:**

Diagnosis of single and co-infection of polyomavirus BK infection in plasma samples is a useful assay to evaluate the risk of BKVAN in kidney transplant patients. Established threshold values for studied viral infections have beneficial use in screening of kidney transplant patients at risk of BKVAN, need to confirm and standardized in completed further studies.

## INTRODUCTION

Human polyomavirus BK (BKV) is a ubiquitous virus causing infection in human. Over 90% of adults worldwide caught polyomavirus BK infection during early childhood [1-6]. A mild respiratory illness in children has been recorded at the time of appearance of antibodies to polyomavirus BK [7, 8]. Following primary infection, the virus can usually persist in the uroepithelial cells, oligodendrocytes, and blood mononuclear cells lifelong [7, 9, 10]. In the large majority of cases, viral infection is silent [7]. Urinary shedding of virus has been detected in 0.5%–20% of asymptomatic population [9, 11]. In those with severe immunological failures such as immunosuppressed transplant patients, latent polyomavirus BK can reactivate [3, 4, 11, 10], leading to unrestricted high viral DNA load in infected tissues and cytolytic destruction of viral target cells [7]. Similar DNA sequences with polyomavirus BK genomic DNA have been observed in renal tissue of healthy normal individuals using hybridization protocols [8]. Polyomavirus BK is as a major cause of kidney allograft dysfunction, cystitis, ureteral stenosis, nephropathy—BK virus-associated nephropathy (BKVAN)—and potential graft loss [4, 11]. Risk factors associated with BKVAN are not known, but introduction of the systematic use of potent immunosuppressive regimens has played an important role [4, 11]. Recipient conditions such as infection or rejection episodes, donor characteristics, such as anti-polyomavirus BK seropositivity and gender, graft features, such as long cold ischemic time can promote polyomavirus BK replication [11]. Its frequency among kidney transplant recipients is usually high (10%–60%) [1, 8, 9, 12]. BKVAN was detected in 1%–10% in renal allograft recipients with loss of renal allograft ranging from 10%–80% [5, 9, 11, 13-15]. Serum creatinine level and urine protein/creatinine ratio (total protein excretion) should be used to screen and follow changes in renal function [16]. So far, no specific antiviral drug is available and the management of BKVAN is merely based on reduction of the immunosuppressive drugs [17]. Immunosuppressive treatment with tacrolimus, mycophenolate, and recently, basiliximab, showed significant associations with the development of detectable polyomavirus BK viremia [11, 17-19]. Before introduction of noninvasive diagnostic tests for polyomavirus BK replication, BKVAN was mostly diagnosed in an advanced stage when irreversible tissue damage had been occurred leading to allograft loss in as many as 90% of transplanted patients [6]. In these patients, histological examination of the allograft biopsy specimens revealed extensive replication of the virus, cell necrosis in the tubules and collecting ducts, and varying degrees of interstitial inflammation [6]. Therefore, early detection of polyomavirus BK replication is feasible and important basic strategy in early diagnosis and treatment of BKVAN to prevent the associated nephropathy [6, 20]. 

Simultaneous co-infection with other viruses can induce BKVAN. Cytomegalovirus-like polymoavirus BK is capable of establishing lifelong latent infection. Reactivation of these viral infections is important cause of post-transplant outcomes. However, the underlying mechanism is not completely clear [21-23].

New molecular methods including PCR and real-time PCR have significantly contributed to the rapid, sensitive, and non-invasive diagnosis of polyomavirus BK [24]. Severity, clinical course, and therapeutic response of BKVAN have all been linked to polyomavirus BK load in urine and blood [25]. Furthermore, viral load determination in blood has significantly been associated with BKVAN [9]. Several studies have demonstrated a correlation between higher viral load in plasma and disease, with positive predictive value ranging from 60% to 85% [4, 11, 12, 25]. Therefore, in this study the frequency of polyomavirus BK infection was evaluated in tissue and blood samples to analyze the possible association between polyomavirus BK infection with post-transplant nephropathy.

## MATERIALS AND METHODS

Patients and Samples

In a cross-sectional study, 108 kidney transplant patients with rising creatinine level >1.5 mg/dL, glomeruli filtration rate (GFR) <30 mL/min/1.73 m^2^ body surface area, and nephropathy symptoms admitted to Namazi Hospital, affiliated to Shiraz University of Medical Sciences, Shiraz, Iran, between 2010 and 2012, were enrolled in this study. The study was approved by the Ethics Committee of Shiraz University of Medical Sciences (the study protocol conformed to the ethical guidelines of the 1975 Declaration of Helsinki). One EDTA-treated blood and one tissue samples were collected from each kidney transplant patient. One tissue sample was also collected from 82 of 108 studied kidney transplant patients. Donors were selected based on ABO blood group compatibility; all of them were negative for cross-matches. The standard conditioning regimen for studied patients included cyclosporine 5 mg/kg initially, a maintenance dose of 2–2.5 mg/kg; cyclosporine level was 50-150 ng/mL; prednisolone 120 mg/day initially, tapering to 10 mg/day; and mycophenolate mofetil 1000 mg/day, twice daily. Acute rejection was initially treated with intravenous steroids. Patients with steroid-resistant rejection were treated with OKT3 [17]. Intravenous acyclovir, 750 mg/day, was administered from day three before transplantation for herpesvirus prophylaxis. A possible association of polyomavirus BK infection with risk factors including pathology results, age, sex, Cellsept, prednisolone, cyclosporine, FK, and creatinine levels and cytomegalovirus infection were evaluated in tissue and plasma samples of studied kidney transplant patients.

Viral Genome Extraction

Polomavirus BK was extracted from plasma and tissue samples using the Invisorb^®^ Spin Virus DNA Blood Mini kit (Invitek, Germany) according to the manufacturer’s instruction. 

Polyomavirus BK Quantitative PCR 

Quantification of polyomavirus BK genome load was done using Genesig BKV real-time PCR (Primer Design Ltd TM, Advanced kit, UK). The standard was prepared using 10-fold dilution of positive control included in the BKV real-time PCR. Polyomavirus BK PCR mix in a final reaction volume of 20 µL containing 5 µL of the DNA, 10 µL Precision™ MasterMix (Applied Biosystems), 1 µL primers and a probe targeting the polyomavirus BK NCCR sequence, 1 µL primers and a probe targeting the internal control (IC) gene, and 3 µL DEPS water. The thermocycling condition for polyomavirus BK included one cycle at 95 °C for 10 min followed by 50 cycles at 95 °C for 5 sec and 60 °C for 60 sec using Step One Plus real-time thermocycler (Applied Biosystems, USA). This quantitative PCR assay was sensitive enough to detect 10 copies of polyomavirus BK genomic DNA per mL of body samples. 

Cytomegalovirus Antigenemia Protocol

Cytomegalovirus antigenemia was performed on EDTA-treated blood samples to evaluate the presence of lower matrix pp65 antigen in polymorph nuclear cells using the CMV Brite Turbo kit (IQ Products, Groningen, Netherlands) according to manufacturer’s instruction as previously described [26]. 

Statistical Analysis

Data were analyzed by SPSS ver 15. χ^2^, Fisher exact test, and Mann-Whitney U test were used to analyze demographic and laboratory indices that may relate to results of polymoavirus BK PCR. A p value <0.05 was considered statistically significant.

## RESULTS

The mean age of 108 (65 male and 43 female) studied kidney transplant patients was 40 (range: 15–68) years.

Polyomavirus BK Infection 

Polyomavirus BK infection was found in 17 (15.7%) of 108 of plasma and 9 (11%) of 82 tissue samples post-kidney transplantation. The polyomavirus BK load in positive plasma samples was >100 copy/mL and <100 copy/mL in tissue samples. The prevalence of polyomavirus BK load in plasma and tissue samples was more elevated in the first year compared with the second and third year post-transplantation follow-up visits. The plasma samples of 6 (5.6%) men and 11 (10.2%) of women of 108 patients were positive for polyomavirus BK infection. The tissue samples of 3 (4%) males and 6 (7%) females of 82 patients studied were found positive for polyomavirus BK infection (Table 1). 

**Table 1:** Risk factors of polyomavirus BK infection in kidney transplant patients suspected to have nephropathy

No	Age (yrs)	Sex	Creatinine (mg/dL)	Cell cept (mg)	Cyclosporine (mg/kg/day)	Tacrolimus (mg/kg/day)	Prednisolone (mg/mL)	Pathology decision	Polyomavirus BK infection in plasma	Polyomavirus BK infection in tissue	Transplant duration (months)	Polyomavirus BK copy number (genome copy/mL)	Cytomegalovirus active infection (PP65 antigenemia)
1	22	Male	7	500	NFD	2	10	Acute cellular rejection grade (ΙΙB), acute tubular necrosis	+^a^	–^a^	9	26600	NFD^b^
2	31	Male	1.7	1500	NFD	3	5	C4d+, no rejection	–	+	11	20	NFD
3	44	Female	1.8	2000	NFD	3	5	Acute tubulointrestitial nephritis	+	–	4	20	NFD
4	20	Female	2.4	2000	NFD	2	2.5	Chronic allograft nephropathy (grade Ι)	–	+	12	20	+^c^
5	15	Male	5.2	1000	100	NFD	5	Acute tubulointrestitial nephritis, chronic allograft nephropathy (grade Ι)	–	+	26	20	NFD
6	33	Male	2.8	1500	NFT	3	25	Acute cellular rejection grade (ΙB)	+	–	11	20	NFD
7	44	Male	1.6	2000	200	NFD	7.5	Acute cellular rejection grade (ΙA), chronic allograft nephropathy (grade Ι)	+	–	34	20	NFD
8	33	Male	8.4	1000	NFT	2	20	Acute cellular rejection (grade ΙΙB)	+	–	28	20	+
9	63	Female	2.3	2000	100	NFD	5	Acute tubulointrestitial nephritis, chronic allograft nephropathy (grade Ι)	–	+	15	40	NFD
10	38	Female	2.5	1500	NFD	3	5	NFD	+	–	18	20	+
11	20	Female	3.3	1500	250	NFD	15	Acute cellular rejection	–	+	15	20	+
12	43	Female	6	2000	NFD	3	5	Acute tubulointrestitial nephritis	–	+	11	20	+
13	42	Female	4	1000	NFD	2	5	Acute cellular rejection (grade ΙA)	+	–	5	120	NFD
14	50	Female	2.8	1000	NFD	1	7.5	Acute cellular rejection	–	+	3	20	NFD
15	27	Male	8	1000	NFD	NFD	5	Acute tubulointrestitial nephritis, acute cellular rejection (grade Ι), chronic allograft nephropathy (grade Ι)	+	–	3	20	NFD
16	45	Male	2.4	2000	NFD	4	5	Acute cellular rejection	Neg	+	13	20	NFD
17	30	Female	2.5	2000	NFD	3	5	Acute cellular rejection grade (ΙΙA), Ab–medated, C4d+ and no rejection	+	–	13	50	NFD
18	36	Female	6.3	2000	NFD	4	5	Acute cellular rejection, acute tubular necrosis	Neg	+	9	20	NFD
19	54	Male	3.5	1000	NFD	2	10	Acute tubulointrestitial nephritis, acute cellular rejection	+	–	20	20	NFD
20	65	Male	3.2	1500	50	NFD	5	NFD	+	NFT	36	40	NFD
21	26	Female	2.8	1500	NFD	3	5	NFD	+	NFT	5	40	NFD
22	51	Male	2.8	2000	125	NFD	5	NFD	+	NFT	5	20	–
23	34	Male	6.4	750	NFD	NFD	5	NFD	+	NFT	60	52	NFD
24	36	Female	1.7	1000	225	NFD	5	NFD	+	NFT	7	20	NFD
25	30	Male	1.7	1500	NFD	2	5	NFD	+	NFT	7	2360	NFD
26	31	Male	1.8	1500	NFD	2	5	NFD	+	NFT	12	62	NFD

a: +=Positive; –=Negative; b: NFD=Data not found, c: +=Rate antigenemia positivity

Cytomegalovirus Co-infection

Active cytomegalovirus infection was found in 13(12%) of 108 transplant patients. Co-infection with cytomegalovirus was found in 2 of 17 plasma and 3 of 9 tissue samples infected with polyomavirus BK (Table 1).

Polyomavirus BK infection and Risk Factors

Associations between various risk factors and infection with polyomavirus BK detected in plasma and tissue samples of kidney transplanted patients are shown in Table 2. Significant associations were found between polyomavirus BK infection in tissue samples with tubulointerstitial nephritis (p=0.001) and acute cellular rejection (p=0.022) (Table 2). There were no significant correlations between polyomavirus BK infection with other pathology results. 

**Table 2:** Association of polyomavirus BK risk factors in kidney transplant patients suspected to have nephropathy

Risk Factors	p value	
	Plasma	Tissue
Sex	0.552	0.135
Age	0.418	0.148
Cellcept	0.225	0.373
Prednisolon	0.622	0.335
Cyclosporine	0.897	0.600
Tacrolimus	0.673	0.455
Creatinine	0.849	0.905
Tubulointerstitial nephritis	0.430	0.001
Acute cellular rejection	0.377	0.022

## DISCUSSION

Polyomavirus BK nephropathy remains important as a post-kidney transplant complication. No clinical risk factors absolutely relates to development of this viral-related nephropathy. Treatment of BKVAN is also problematic and no antiviral medication is approved. Monitoring anti-polyomavirus BK specific immunity is also not widely available. Therefore, molecular screening of polyomavirus BK replication and targeted reduction of immunosuppression can resolve infection and improve renal function by stabilizing serum creatinine in kidney transplanted patients [12, 27]. From patient samples, serial screening of plasma polyomavirus BK load is a valid tool to identify patients at risk of BKVAN [4]. Such finding becomes even more relevant because BK viremia only occurs under active replication not being found during latent infections [11, 12, 17]. However, researchers recently reported marked variability between commonly used polyomavirus BK load assays [28, 29]. Furthermore, neither polyomavirus BK real-time PCR nor the cut-off value for significant viral replication has been standardized [6]. Therefore, a threshold value should be established for each different quantitative polyomavirus to clearly screen BKVAN.

In this study the polyomavirus BK infection was studied in kidney transplanted patients with elevated level of creatinine and risk of BKVAN. Polyomavirus BK load was found in 15.7% of plasma and 11% of tissue samples. The polyomavirus BK load was >100 copy/mL in plasma and <100 copy/mL in tissue samples. Also polyomavirus BK load in plasma and tissue samples was more elevated in the first year compared with the second and third year post-transplantation follow-up visits. In a study, the rate of BKVAN was found in 36 (2%) of 1788 kidney transplant patients [9, 29, 30]. In other single-center reports the rate of polyomavirus BK load was 7.5% [6, 30]. Similarly in another report the polyomavirus BK load was found in 7 (35%) of 26 transplanted recipients [4]. A retrospective analysis of urine and plasma samples of 30 kidney recipients found that eight (27%) patients were positive for polyomavirus BK viruria [13]. The overall prevalence of polyomavirus BK DNAuria and DNAemia were 40.7% and 9.2% in 76 studied transplant patients, respectively [14]. In another retrospective cohort, the rate of BKVAN was 3.7% [11]. The plasma PCR was superior to urine PCR or cytology in specificity and positive predictive value for detection of BKVAN. Regular monitoring of plasma PCR detected significant polyomavirus BK viremia in 8.3% of patients [12]. Plasma PCR is useful in predicting an increased risk for BKVAN [6]. In another investigation, polyomavirus BK viremia was found in 43.3% of kidney recipients [28]. However, in controversy with this report and other earlier studies, Hammarin, *et al*, was not found any patients with BKVAN and any transplanted patients with a permanent deterioration of graft function. After six years of monitoring these kidney transplanted patients, it was clearly shown that patients with low viral loads were often intermittently became positive for polyomavirus BK DNAmia for long periods and this viremia has no clinical relevance in the majority of patients [17].

In their study, 8.3% of patients were positive for Epstein-Barr virus. Moreover, Nada, *et al*, reported coexistence of cytomegalovirus and BKV infections in a biopsy specimen from a patient who experienced acute rejection [8]. From these reports, it may be suggested that cytomegalovirus infection is associated with BKV infection, and *vice versa*, which differs from results of the present study. However, all of these studies reported co-infection with these two viruses, whereas the present study examined only the coexistence of these viruses. Therefore, positivity for one virus does not necessarily mean acute infection.

Several risk factors have been associated with increased polyomavirus BK replication and nephropathy progression, including warm ischemia and reperfusion injury, cytomegalovirus co-infection, the level of panel reactive antibodies, duration of dialysis, and the type of immunosuppressive therapy [31, 32]. Cytomegalovirus and polymoavirus BK co-infection may have role on promoting or limiting their related pathogenesis [21-23]. Polyomavirus BK virus may induce the expression of cytomegalovirus genes by stimulating cellular regulator proteins and/or by its related gene regulator proteins [21-23]. Coexistence of these viral infections has been reported with controversy in kidney transplant recipients with allograft nephropathy [22, 23]. In this study, also 11.8% and 33.3% of plasma and tissue samples of kidney transplanted recipients were simultaneously co-infected with polyomavirus BK.

However, it remains unclear whether specific immunosuppressive agents or their respective doses are critical to BKVN development [32]. In some studies higher viruria was found in treatment regimens combined of tacrolimus and mycophenolate mofetil compared with patients treated with cyclosporine and mycophenolate mofetil [9, 11, 32]. However, in this study significant association was found only between viral load in tissue samples with two pathology results tubulointerstitial nephritis and with acute cellular rejection. Similar to some reports [29], significant associations were not seen between polyomavirus BK infection and other risk factors including age, sex, use of Cellsept, prednisolone, and cyclosporine, FK, and creatinine levels. Similar to another report [6], in this study cytomegalovirus co-infection was found in only one patient with polyomavirus BK infection.

In conclusion, based on these results, measurement of polyomavirus BK replication in plasma in comparison with tissue samples is a valuable assay to evaluate the risk of BKVAN in kidney transplant patients. Established threshold value, which has beneficial use in screening of Iranian kidney transplant patients at risk of polyomavirus BK-related nephropathy in Iranian kidney transplant patients, need to confirm and standardized in completed further studies.
